# Availability of emergency contraception in large Brazilian municipalities: a guaranteed right?

**DOI:** 10.3389/fphar.2023.1023464

**Published:** 2023-11-27

**Authors:** Ana Carolina Gomes Pinheiro, Bárbara Manuella Cardoso Sodré Alves, Claudia Mara Pedrosa, Tiago Marques dos Reis, Andréa Dâmaso Bertoldi, Ivan Ricardo Zimmermann, Silvana Nair Leite, Rafael Santos Santana

**Affiliations:** ^1^ Faculty of Health Sciences, University of Brasília (UnB), Brasília, Federal District, Brazil; ^2^ Faculty of Pharmaceutical Sciences, Federal University of Alfenas (Unifal), Alfenas, Minas Gerais, Brazil; ^3^ Post-Graduation Program in Epidemiology, Federal University of Pelotas (UFPEL), Pelotas, Rio Grande do Sul, Brazil; ^4^ Department of Pharmaceutical Sciences, University of Santa Catarina (UFSC), Florianópolis, Santa Caratina, Brazil

**Keywords:** emergency contraception, unified health system, prescriptions, women's health, Brazil

## Abstract

**Introduction:** Emergency Contraception (EC) is available in Brazil since 1996, when it was adopted as one if the family planning strategies and, in 1998, for use in services assisting victims of sexual violence. In the country, its use is regulated by guidelines. Its access through SUS (Unified Health System), however, does not seem to occur in a standardized manner.

**Methods:** The aim of the study was to analyze the availability and barriers to accessing emergency contraception (levonorgestrel) in Brazilian municipalities with more than 500 thousand inhabitants. The survey was carried out by a form sent to the Municipal Health Departments (SMS) managers and a search on the list of standardized medicines by the hospitals in the same municipalities.

**Results:** The Basic Health Units were identified as the standard access places to EC. However, one of the obstacles mentioned is the need for a prescription for dispensing in almost 80% of the analyzed cities. Access in emergency situations at night and on weekends is also uncertain, since although 67% of the places stated that they dispense at the hospital level, the item was only standardized in 21% of the hospital lists.

**Discussion:** The difficult access this drug in the public system essentially tends to harm the poorest women, who are the ones who suffer most from the consequences of an unwanted pregnancy.

## 1 Introduction

Emergency Contraception (EC), commonly known as the morning-after pill, has established itself over the years as an essential contraceptive method in guaranteeing women’s sexual and reproductive rights. Its main objective is to help prevent unwanted pregnancies after sexual intercourse, whether due to cases of violence, unprotected sex or failure of the routine contraceptive method (Cavalcante, 2009; Brazilian Ministry of Health, 2011). However, society’s misinformation on the risks and benefits of the pill, results in legal challenges regarding its sale and distribution in several countries around the world (International Consortium for Emergency Contraception ICEC, 2018; International Consortium for Emergency Contraception ICEC, 2021).

In Brazil, Levonorgestrel is the medicine dispensed free of charge by the Unified Health System (SUS) for EC (Brazilian Ministry of Health, 2012). Financing and procurement are carried out by the Ministry of Health under the Family Planning and Women’s Health Policies (Brazilian Ministry of Health, 2004; Brazilian Ministry of Health, 2016; Pizzol et al., 2016). It is also possible to be acquired through private pharmacies, which is the population’s main access route, since in practice it is accessed without a prescription (Figueiredo, 2016).

The Protocol for the Use of Levonorgestrel in Emergency Hormone Contraception was launched in 2012 by the Ministry of Health, with the goal of expanding informational tools on this therapy. In the document, there is a provision for free of charge dispensation by SUS without the need for a medical prescription, also delegating care to the nurses (Brazilian Ministry of Health, 2012; Dos Santos et al., 2014).

The use of EC has grown over the years and now ranks fifth among the methods used by most women. According to the National Demographic and Health Survey (PNDS), 12% of women said they had used the method at least once in their lifetime (Pesquisa Nacional de Demografia, 2009; Brandão, 2017). Data from the National Survey on Access, Use and Promotion of the Rational Use of Medicines (Pnaum) also show that the general use prevalence of oral contraceptives in the country is of almost 30% of the female population of childbearing age. However, many Brazilian women obtain this medicine directly from commercial pharmacies (78%), even though it is available through SUS (Farias et al., 2016). The private sector is also a reference in dispensing this method in Sub-Saharan Africa countries, while it is in public-funded health facilities that EC are most available in urban centers in India (Muhoza et al., 2021).

It is estimated that about 25% of Brazilian women experience unplanned pregnancies, however, there is no accurate data on the prevalence of abortions in Brazil. In England, 30% of pregnancies are unwanted, and 20% of all pregnancies end in abortion. In the United States, almost 50% of pregnancies are unwanted, and 40% of these result in abortion (Figueiredo and Bastos, 2008; Cardoso et al., 2020; Burke, 2022).

Even though emergency contraception is already consolidated as a method of family planning policies, a study conducted in the Northeast region of Brazil revealed that even though 85.5% of professionals prescribe the method, only 8.5% of them actually consider it a woman’s right (Spinelli MBA da et al., 2014; Muhoza et al., 2021). Another study carried out in the federal capital demonstrated failures in supplying EC and in the information provided about the method (Figueiredo, 2016; Instituto Brasileiro de Geografia e Estatística IBGE, 2021). In São Paulo, the most populous state in the country, it was observed that the distribution and organization of access in the different municipalities of the state do not follow a pattern and present different availability results according to the level of local structure (Helena et al., 2007).

Given this scenario, this study aimed to analyze the availability of emergency contraception in public-funded health facilities in the largest Brazilian 43 municipalities (with more than 500 thousand inhabitants) and to identify the potential barriers to access the levonorgestrel in the country.

## 2 Methods

This is an exploratory study carried out between October 2019 and March 2020. Initially, Brazilian municipalities with over 500 thousand inhabitants were identified through census data from the Brazilian Institute of Geography and Statistics (IBGE) (Instituto Brasileiro de Geografia e Estatística IBGE, 2021). All Municipal Health Secretariats (SMS) were contacted to participate in the study and provide public data, based on Law No. 12,527/2011, known as the Access to Information Law (LAI), which regulates the constitutional right of access to government data (Brasil. Portal do Cidadão, 2021).

The collection of data was conducted through a research instrument with questions prepared by the researchers that was sent through the official communication channels of the secretariats (ombudsman). This instrument consisted of a questionnaire with 8 questions, which dealt with the availability of emergency contraception in the municipality’s primary healthcare centers and the potential barriers to accessing this method by women who are SUS users, as described below:

I. What are the access points to levonorgestrel through SUS in the municipality?
II. Are there any SUS dispensing points on weekends or on a 24-h schedule?
III. What is required to have access to levonorgestrel through SUS?
IV. Can dispensation be carried out without a medical prescription?
V. Must the prescription be from SUS or can it be of private origin?
VI. Can dispensation be performed with the prescription of another health professional?
VII. Can the Delivery/Dispensation be done to the user’s partner or caregiver?
VIII. Do you follow any specific protocol and/or standard for dispensing EC?

The collected data was analyzed using descriptive statistics per municipality analyzed. The answers to the questions “*Are there any SUS dispensing points on weekends or on a 24-h schedule?*”, “*Can dispensation be carried out without a medical prescription?*” and “*Must the prescription be from SUS or can it be of private origin*?” were categorized, for analysis, as: “Yes”, “No”, or “Inaccurate” (when the answer was not clear even after a new request for clarification).

The questions *“What are the access points to levonorgestrel through SUS in the municipality?”* and “*What is required have access to levonorgestrel through SUS?*” were analyzed separately, and categorized according to their similarity and context.

Finally, for the question “*Do you follow any specific protocol and/or standard for dispensing emergency contraception?”*, in addition to being categorized into “Yes” and “No”, a survey of which protocols and/or standards are used by the municipalities was also carried out according to the information obtained.

Additionally, the standardization lists of available medicines from the largest hospitals in these municipalities were consulted to check whether levonorgestrel was listed as an item. For this purpose, medication lists were searched on the official websites of hospitals or health departments. The lists were retrieved from the institutional websites of the hospitals/secretariats or requested to the referred entity when unavailable.

For the selection of units, the National Register of Health Establishments (CNES) was consulted, and the largest hospital in terms of the number of beds under federal, state, and municipal management in the evaluated city (when available) was selected. The specific searches for this stage were conducted from January to March 2020 through the following web address: https://cnes.datasus.gov.br/pages/consultas.jsp

## 3 Results

### 3.1 Locations with access to emergency contraception in SUS

Fifty-two municipalities that met the established inclusion criteria were identified. Of these, 43 (83%) agreed to participate in the survey by answering the invitation made by telephone and the questionnaire sent. Only 17% (nine) of the contacted municipalities did not return the information: i) Palmas-TO, ii) Campos dos Goytacazes-RJ, iii) Macapá-AP, iv) Ananindeua-PA, v) Aparecida de Goiânia-GO, vi) São Luis-MA, vii) Goiania-GO, (viii) Manaus-AM, ix) Natal-RN.

It is noteworthy that, according to IBGE (Brazilian Institute of Geography and Statistics) data (Instituto Brasileiro de Geografia e Estatística IBGE, 2010), the female population in Brazil is of 109,4 million in 2019 and the present study, through the investigated municipalities, covered about 29% of this population.

Of the municipalities interviewed, all perform EC dispensation, and declare that the main place of access to EC is via the Primary Healthcare Centers (UBS). Some SMSs even mentioned locations such as Emergency Care Units (UPAs), hospitals and municipal pharmacies as dispensing locations (Table 1).

**TABLE 1:** Locations with access to Emergency Contraception in Brazilian capitals and municipalities with more than 500 thousand inhabitants (2019/2020).

Region (number of cities)	Brazil (*n* = 43)	Northeast (*n* = 9)	North (*n* = 4)	Midwest (*n* = 3)	Southeast (*n* = 21)	South (*n* = 6)
Primary care units	43 (100%)	9	4	3	21	6
Emergency care units and Hospitals	29 (67%)	5	2	2	15	5
Municipal Pharmacies	3 (7%)	-	-	-	2	1
Others^a^	3 (7%)	1	-	-	1	1

^a^ Maternity wards, the Department of Women, Children and Adolescents, polyclinics.

Nonetheless, the non-availability of the method on 24-h schedules and on weekends presents barriers to its access, since the vast majority of UBSs generally follow business hours (from Monday to Friday) (Brazilian Ministry of Health, 2013). Given this fact, it is possible to verify that 33% of the municipalities admit that they do not have any EC dispensing points working on 24-h schedules (UPAs and Hospitals), free of charge and with available guidance.

It is necessary to emphasize that, although almost 70% of managers stated that they guarantee access to EC at any time in the hospital network, that was not found when consulting the medicines lists of the largest hospitals in these municipalities. In the 43 cities analyzed, 67 lists of hospitals were obtained, and it was verified that only 15 hospitals (19%) of 15 cities (34%) described levonorgestrel 750 mcg or 1.5 mg in their medicine’s lists.

This apparent disagreement in the information may indicate that the signaling of institutional access may not be observed in practice, and that especially women who have suffered sexual violence may not be adequately taken care regarding of pregnancy prevention in the largest Brazilian public hospitals.

### 3.2 The practice of dispensing emergency contraception

**FIGURE 1:**
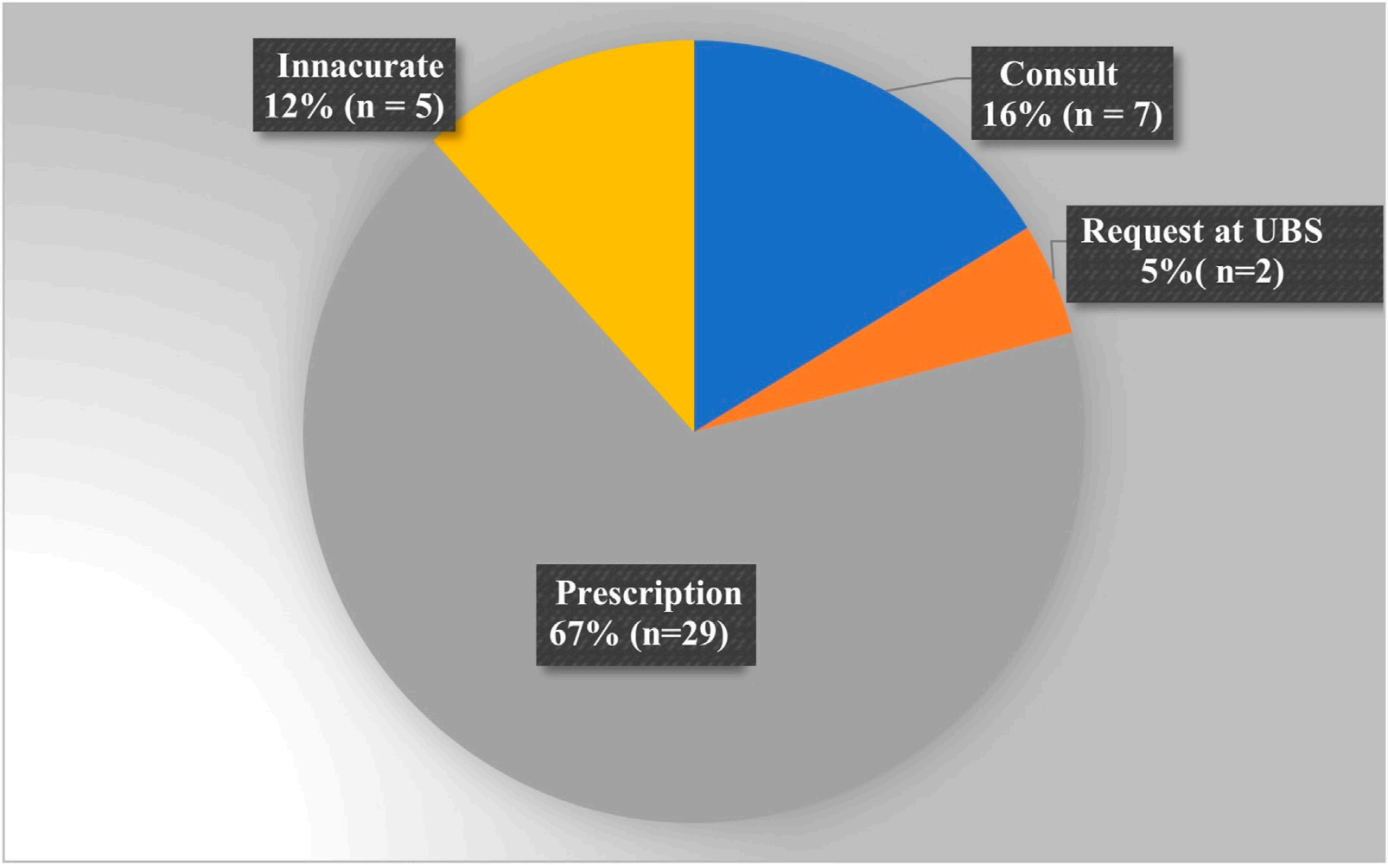
Necessary requirements to obtain emergency contraception, according to the municipalities evaluated.

Most municipalities (67%) reported that prescription was essential for the access to levonorgestrel (Figure 1).

The main access barriers identified through questions to municipal management are described in Table 2 and demonstrate the variability of pattern of care among the largest Brazilian municipalities.

**TABLE 2:** Assessed capitals and municipalities with more than 500 thousand inhabitants and potential barriers to access emergency contraception in 2019/2020.

Municipality	Available in municipality’s primary healthcare centers?	Available 24 h facilities? (According to management)	Flexible prescription (medical or other)	Access through caregiver	Available in hospitals? (According to list)	Access without prescription
Aracaju (SE)	Yes	Yes	Yes	Yes	No	No
Belém (PA)	Yes	Yes	Yes	No	No	No
Belford Roxo (RJ)	Yes	No	No	Yes	No	No
Belo Horizonte (MG)	Yes	Yes	Yes	-	Yes	Yes
Boa Vista (RR)	Yes	Yes	Yes	No	Yes	No
Brasília (DF)	Yes	No	Yes	Yes	No	No
Campinas (SP)	Yes	Yes	Yes	No	No	No
Campo Grande (MS)	Yes	Yes	Yes	Yes	Yes	No
Caxias do Sul (RJ)	Yes	No	Yes	No	No	No
Contagem (MG)	Yes	Yes	Yes	Yes	No	No
Cuiabá (MT)	Yes	Yes	Yes	No	Yes	No
Curitiba (PR)	Yes	Yes	Yes	Yes	Yes	No
Duque de Caxias (RJ)	Yes	Yes	No	Yes	No	No
Feira de Santana (BA)	Yes	-	Yes	Yes	-	No
Florianópolis (SC)	Yes	Yes	Yes	Yes	Yes	No
Fortaleza (CE)	Yes	No	Yes	Yes	Yes	No
Guarulhos (SP)	Yes	Yes	Yes	Yes	-	-
Jaboatão dos Guararapes (PE)	Yes	No	No	No	No	No
João Pessoa (PB)	Yes	Yes	Yes	Yes	No	No
Joinville (SC)	Yes	Yes	Yes	Yes		Yes
Juiz de Fora (MG)	Yes	No	No	Yes	No	No
Londrina (PA)	Yes	Yes	Yes	Yes		Yes
Maceió (AL)	Yes	No	Yes	-	No	No
Niterói (RJ)	Yes	Yes	No	Yes	No	No
Nova Iguaçu (RJ)	Yes	Yes	Yes	Yes	-	No
Osasco (SP)	Yes	Yes	Yes	Yes	-	No
Porto Alegre (RS)	Yes	Yes	Yes	Yes	Yes	No
Porto Velho (RO)	Yes	No	-	No	No	Yes
Recife (PE)	Yes	Yes	Yes	Yes	Yes	No
Ribeirão Preto (SP)	Yes	No	Yes	-	No	No
Rio Branco (AC)	Yes	No	Yes	Yes	No	No
Rio de Janeiro (RJ)	Yes	Yes	Yes	Yes	Yes	No
Salvador (BA)	Yes	Yes	Yes	Yes	Yes	No
Santo André (SP)	Yes	No	Yes	Yes	-	No
São Bernardo do Campo (SP)	Yes	No	Yes	Yes	-	Yes
São Gonçalo	Yes	Yes	No	Yes	No	No
São José dos Campos (SP)	Yes	No	Yes	Yes	No	Yes
São Paulo (SP)	Yes	Yes	Yes	Yes	Yes	No
Serra (ES)	Yes	Yes	Yes	Yes	-	Yes
Sorocaba (SP)	Yes	Yes	Yes	Yes	-	No
Teresina (PI)	Yes	Yes	Yes	No	Yes	Yes
Uberlândia (MG)	Yes	Yes	-	No	No	No
Vitória (ES)	Yes	Yes	Yes	Yes	Yes	Yes
TOTAL	Yes = 100%	Yes = 67%	Yes = 81,4%	Yes = 72%	Yes = 34%	Yes = 21%

Even though this is a product distributed nationally by acquisition of the federal entity, due to the unavailability of national public data, this study was not able to obtain distribution data from most of the evaluated municipalities. However, in some of these municipalities, it is possible to obtain indications that somewhat restrictive actions can influence the access.

Among the municipalities with more flexible access, there was the dispensation, in the last year, of approximately 553 treatments per 10,000 women of childbearing age in Belo Horizonte (MG) and of 581 treatments per 10,000 women of childbearing age in Florianópolis (SC). Among the municipalities with stricter access rules, it was observed 72 treatments per 10,000 women of childbearing age in Jaboatão dos Guararapes (PE) or 65 treatments per 10,000 women of childbearing age in Brasília (DF). Different local characteristics can still influence these results, such as the degree of coverage of primary care or local organization of pharmaceutical care and available dispensing locations.

## 4 Discussion

Whereas in Brazil the use of EC based on oral levonorgestrel pills has been regulated in Family Planning actions by the Ministry of Health since 1996, and for the care of victims of violence in health services since 1998 (Figueiredo, 2016), its use, despite all guidelines, remains non-standardized, a fact highlighted by several authors over the years (Hardy et al., 2001; Osis et al., 2006; Souza and Brandão, 2009). The progress made in regard to EC standardization by the Ministry of Health is extremely important, however, they are not sufficient to guarantee easy and safe access to the Brazilian population, and consequently the integrality of care for women’s health problems and prevention of pregnancy resulting from violence against women. This change is becoming more urgent, as Law 9263 has established for almost 3 decades that all three instances of the Unified Health System (SUS) must ensure, among other actions, full access to contraceptive methods, whether they are permanent or temporary, such as emergency contraceptives. (Souza and Brandão, 2009).

According to Bastos et al., quick access to EC ensures greater efficacy, especially on weekends or at night, when, in general, the contraceptive is most needed (Figueiredo, 2016). The fact of not having this access facilitated by the public health service can generate an increase in demand in private places, since most commercial pharmacies have extended opening hours or are open 24 h a day. For these reasons, contraceptives in general have a tendency to be acquired in private services, most of the time, by direct disbursement by the user; Even in the poorest regions of Brazil, such as the northern region, up to 81% of Brazilian women pay for their contraceptives, which may indicate important access barriers, especially in situations of emergency use. (Pesquisa Nacional de Demografia, 2009; Dos Santos et al., 2014)

For Costa et al., (Hardy et al., 2001) the mandatory medical prescription in health services in Brazil is also one of the main barriers to accessing EC. The requirement of medical or nursing consultation to obtain a prescription impedes many women from acquiring the method in these services (Costa et al., 2008; Figueiredo, 2016). In the other hand, it is important to highlight that the professional should also take this access to EC as an crucial opportunity to make a long-term contraception establishment. According to the International Consortium for Emergency Contraception, until January 2020, 80 countries had permission to purchase the method without the need for a medical prescription (International Consortium for Emergency Contraception ICEC, 2021). Although the medical consultation is undoubtedly important in most cases of medicine use, in this case, the non-mandatory requirement, associated with the improvement of dispensing practices for correct guidance and identification of warning signs, tends to be related to a reduction in the number of unintended pregnancies and unsafe abortions. This fact is relevant for women who really cannot or do not want to become pregnant, especially in countries where abortion is not allowed by law, which is the case for Brazil (Oliveira and Oliveira, 2016).

Since 2015, two EC methods have been available in Germany as over-the-counter emergency contraceptives. The basis for this decision was the existing evidence that EC delays ovulation and, therefore, the occurrence of an unwanted pregnancy can be prevented, in addition to both having a good safety profile. After analyzing the available data on EC, the author shows that with easier access, more women use the method after unprotected sexual intercourse. However, it is noteworthy that, despite the growth in the market, EC is very rarely used after unprotected sex in Germany. This is shown by comparative data for other European countries, estimates of the frequency of unprotected sex and numbers on unwanted pregnancies (Kiechle and Neuenfeldt, 2017).

It is noteworthy that the requirement of a medical prescription for access to EC, despite hampering free access via SUS, in practice, does not prevent the acquisition of the method in the private system, which normally dispenses EC without any document requirement (Bastos et al., 2009; Paiva and Brandão, 2014; Figueiredo, 2016). Therefore, another possibility of EC distribution in SUS is to promote direct prescription or dispensing by other health professionals, such as nurses and pharmacists, especially when there are no doctors available in the unit (Brazilian Ministry of Health, 2012; Figueiredo, 2016), this measure can expand access and ensure correct orientation in regard to EC use. A survey carried out in São Paulo, Brazil’s largest city, showed that 94.2 per cent of people who reported having used emergency contraceptives bought them at a private pharmacy, without presenting a prescription. [30]The Brazilian Ministry of Health’s Protocol for the use of Levonorgestrel already allows, in primary healthcare, the dispensing of EC without the need for a medical prescription. Due to the large number of municipalities that do not allow such dispensing, it was questioned whether it was possible to dispense it with the indication and/or prescription made by another health professional, since the protocol for using levonorgestrel states: “For the dispensing of levonorgestrel, no medical prescription will be required, and the nurses may provide the EC in the absence of the doctor and subsequently refer the user to reproductive planning actions” (Brazilian Ministry of Health, 2012). Article 11, item II, § 3 of Law no. 7498/86, regulates the nurse’s right to prescribe medicines previously established in public health programs within routines defined and approved by the institution (Jusbrasil, 1986; Brazilian Ministry of Health, 2011).

According to a study carried out by Figueiredo et al., the presence of the Nursing team in the UBSs of the cities of São Paulo, in the process of dispensing the EC, is poorly utilized, considering that in 55.3% of the cities in the study professionals in this area do not dispense the method (Helena et al., 2007). This fact could be confirmed by the present study, since the non-participation of other professionals in dispensing the method is still very common. Most municipalities (81.4%) stated that dispensing is allowed upon prescription and/or indication of a professional other than the doctor.

It is inferred, from this scenario, the importance and necessity of creating updated protocols and measures, to increasingly help the insertion, not only of nurses, but also of pharmacists as professionals of dispensing the method throughout the country. As an example, in Australia, since 2004, EC is dispensed through community pharmacies as an over-the-counter medicine (Hussainy et al., 2015).

It is also urgent to highlight the need for access also through users caregivers, as several factors lead women to resort mainly to their partners to obtain the EC when they observe failure of the protection method previously chosen or even in an unprotected intercourse. The feeling of shame and embarrassment for using the method also encourages access to be made by third parties, since, according to a study by Paiva and Brandão, many consumers were considered “shameless” and discriminated against when purchasing the method when they did not demonstrate a posture of evident regret (Paiva and Brandão, 2017).

Furthermore, in cases of violence, in addition to shame or fear of exposing the situation (Monteiro and Zaluar, 2012; Drezett, 2016), a study revealed that the fear of revictimization within institutions or of reprisals are pointed out as relevant reasons for women’s resistance in searching clinical care (Silva et al., 2019).

In SUS, as a rule, the withdrawal of a medicine is usually done by means of a prescription and by showing an identification document with photo. If it is done by a third party, the identification of the caregiver is also required and, in many places, this is allowed, given the impossibility of access for some patients due to physical or transport limitations. However, in this study, almost 30% of the evaluated locations said they do not allow such act, as they claim that the patient’s presence is essential, which makes access through SUS even more difficult and consequently leads the partner and/or caregiver to seek the method in private establishments (Silva et al., 2019).

The way in which the EC has been dispensed reveals numerous differences in its process across the country, even with the availability of documents such as the “Protocol for the Use of Levonorgestrel”, “Emergency Contraception: question and answers for health professionals” and the “Notebook of Women’s Health” (Brazilian Ministry of Health, 2011; Brazilian Ministry of Health, 2012; Brazilian Ministry of Health, 2016). These documents are intended to assist in the dispensing of EC and to resolve any doubts regarding the method and, consequently, help standardize the process across the country. When questioned during the study, 75% of the municipalities confirmed adopting the national protocols but showed conflicts, unfamiliarity, or non-compliance with the guidelines in the other questions answered. Another 25% reported not following dispensing protocols or did not respond.

Most municipalities, when asked about the existence of standardization or guidance for dispensing EC, claimed to use some type of protocol. Even with a favorable scenario in which most municipalities claim to follow some protocol, the amount of various documents used to carry out this practice is an alarming factor, given that it directly contributes to the difficulty of implementing unified practices that enable the dispensation method in a continuous and orderly manner throughout the country, to guarantee the right of access for all women.

The low number of standardized emergency contraceptives (EC) in hospital lists (34%) is undoubtedly a concerning fact, considering that it is already established in Brazil by the “Lei do Minuto Seguinte” (Law 12845) that “pregnancy prophylaxis and prophylaxis of Sexually Transmitted Diseases - STDs” are mandatory in all hospitals belonging to the SUS network. Therefore, it was expected that the access would be close to 100%, especially in large cities’ hospitals. This result raises doubts that may be the subject of further investigations, such as the preparedness of the healthcare teams in these units and the provision of other equally mandatory actions, such as psychological and social support, monitoring exams, or measures for identifying and punishing offenders.

It is important to mention that the limitations of this study can constrain a broader understand of the phenomenon of the access and use of EC in Brazil. Difficulty in accessing some public information was a limitation of this study, other research that bring data from users’ attempts and therapeutic itinerary are recommended. Data on the number of dispensed medications were also obtained; however, due to their inaccuracy and uncertainty of the results, it was decided not to include them in the study. It is also important to emphasize that the study chose to evaluate only large cities, and therefore, in small cities with less structured pharmaceutical service processes, there may be additional access difficulties. Further research may contribute to this regard.

## 5 Final considerations

The findings demonstrate that barriers to accessing EC in public health services are still very prevalent and that there is an urgent need for reorganization and improvement of practices for dispensing the EC to ensure full access to prevention of unwanted pregnancy, given the huge discrepancy reported concerning the process in the country. Similarly, even though the data is already poor, as it was obtained through the public information access law, it may have suffered from response softening bias, and in practice, the access barriers may be even greater.

Exemption of the mandatory prescription or encouragement of prescription by other health professionals, review of clear access rules, expansion and dissemination of dispensing facilities seem to be measures that should be evaluated by SUS managers so that it is possible to expand the access of women and couples to EC and to offer post-sexual violence care.

## Data Availability

The original contributions presented in the study are included in the article/supplementary material, further inquiries can be directed to the corresponding author.
